# *Misi Yehewin* (big breath): a cross-sectional survey series of Métis health and wellbeing during the early COVID-19 pandemic in Alberta, Canada

**DOI:** 10.3389/fpubh.2025.1741161

**Published:** 2026-01-13

**Authors:** Maria B. Ospina, Reagan Bartel, Jesus Serrano-Lomelin, Sana Amjad, Ashton Anderson, Ian Colman

**Affiliations:** 1 Department of Obstetrics and Gynecology, University of Alberta, Edmonton, AB, Canada; 2 Department of Public Health Sciences, Queen’s University, Kingston, ON, Canada; 3 Otipemisiwak Métis Government of the Métis Nation within Alberta, Edmonton, AB, Canada; 4 School of Epidemiology and Public Health, University of Ottawa, Ottawa, ON, Canada

**Keywords:** COVID-19, indigenous, mental health, Métis (North American people), minority health, wellness

## Abstract

**Introduction:**

The COVID-19 pandemic disproportionately affected Indigenous populations, yet Métis-specific data remain limited. We described COVID-19-related experiences, physical and mental health, health behaviours, and socio-economic wellbeing among Métis people in Alberta (Canada) during the early pandemic.

**Methods:**

*Misi Yehewin* was a cross-sectional survey series conducted with the Otipemisiwak Métis Government of the Métis Nation within Alberta. Self-identified Métis aged ≥16 years completed surveys in three phases: December 2020–January 2021 (Wave 1), March–April 2021 (Wave 2), and November–December 2021 (Wave 3). Each wave included an independent sample of participants. We calculated weighted proportions for 28 key items and compared estimates across waves.

**Results:**

Overall, 2,439 participants completed the surveys. Confirmed COVID-19 cases were reported by 5% of participants in Wave 1 and 15% in Wave 3. Reports of worsening physical and mental health were less frequent in later phases; yet, across waves, 41% screened positive for depressive symptoms, 47% for anxiety, and 68% for high perceived stress. Food insecurity was reported by 39.4% of participants in Wave 1 and 52.9% in Wave 3. Reduced family time and cultural activities were common, particularly in earlier waves. Reports of financial strain (~56%), racism (~25%), and strong Métis identity (~89%) was similar across waves.

**Conclusion:**

Findings highlight ongoing structural inequities influencing Métis health during COVID-19. Despite fewer reports of worsening overall health in later phases, symptom-based measures showed persistently high perceived stress and widespread food insecurity. Métis-led, culturally grounded strategies are needed to address both immediate and long-term determinants of health.

## 1 Introduction

The years 2020–21 marked the early and most intense phases of the COVID-19 pandemic, characterized by widespread disruption and profound health impacts worldwide. However, the burden of the pandemic was not evenly distributed across communities, regions, and population groups. Certain populations, particularly those affected by the social and historical consequences of colonialism, faced heightened risks of infection, barriers to healthcare access, and deepened existing inequities (1–3).

Indigenous peoples have long faced disproportionate health inequities both globally (4) and in Canada, including the Métis (5, 6). These inequities have been magnified during public health crises such as the 2009 H1N1 influenza outbreak (7) and the COVID-19 pandemic. Despite Canada’s universal healthcare system, Indigenous communities have often been overlooked in pandemic assessments and responses. During the H1N1 outbreak, Indigenous peoples experienced significantly higher rates of infection, hospitalization, and severe outcomes compared to non-Indigenous populations. For example, the infection rate among the Inuit reached 1,000 per 100,000, compared to 24 per 100,000 among non-Indigenous individuals in Canada (8). The Public Health Agency of Canada reported that Indigenous people accounted for 12.6% of confirmed H1N1 cases, 17% of hospitalizations, and 14% of intensive care unit admissions, despite representing a much smaller proportion of the population (~5%) (9, 10). The COVID-19 pandemic reinforced these disparities, emphasizing the urgent need for proactive planning, enhanced pandemic preparedness and equitable response strategies to reduce the disproportionate burden on Indigenous communities and support more inclusive public health decision-making.

Yet, Métis-specific experiences during COVID-19 remain under-documented. As one of Canada’s three constitutionally recognized Indigenous groups, the Métis are often referred to as the “hidden” Indigenous peoples due to the persistent lack of research, policies, and services tailored to their unique needs and experiences (11). Recent Métis-specific studies have begun to document pandemic experiences using qualitative methods (12–15) and administrative data, but these do not provide community-based survey data on multiple health, social, cultural, and economic outcomes for Métis people. Little is known from a community-based survey perspective, about Métis people’s health, economic security, cultural continuity, and kinship networks during the COVID-19 pandemic.

*Misi Yehewin,* meaning “big breath” in Michif, the traditional language of the Métis, symbolizes a moment to pause and reflect, especially during times of great uncertainty such as the COVID-19 pandemic. In light of these gaps, this study aimed to describe COVID-19-related experiences among Métis people in Alberta during the early phases of the pandemic, and to examine differences in physical and mental health, health behaviours, and social and economic wellbeing across three different phases of the early pandemic. Using data from a cross-sectional survey series, we estimated proportions of key outcomes and explored whether there were sample-level differences in these outcomes across the three survey waves during the evolving public health context of the pandemic.

## 2 Methods

### 2.1 Context and setting

This study was conducted in Alberta, a western Canadian province of ~4.8 million people, including 127,475 self-identified Métis: 20.4% of Métis people in Canada (16). The Otipemisiwak Métis Government of the Métis Nation within Alberta (MNA) represents over 70,000 citizens and is Canada’s largest Métis governance body (16).

The MNA played a central role in the study design, survey implementation, and interpretation of results. This partnership was formalized through a research agreement with a team comprising Métis, MNA representatives, and academic researchers. The study was approved by the University of Alberta Health Research Ethics Board (Pro00104745) and followed the six principles of ethical Métis research outlined by the former Métis Centre of the National Aboriginal Health Organization (17).

### 2.2 Study design

We conducted a cross-sectional web-based survey series in three waves from December 2020 to December 2021, aligned with distinct phases of the early COVID-19 pandemic in Alberta. The study adheres to the Checklist for RepOrting Survey Studies (CROSS) (18), and the CHecklist for Reporting Results of Internet E-Surveys (CHERRIES) (19). The first survey (December 18, 2020–January 31, 2021) was conducted during Alberta’s second COVID-19 wave, a period of province-wide lockdowns, gathering restrictions, and limited vaccine availability. The second survey (March 20–April 30, 2021) coincided with the third pandemic wave, marked by high case rates, renewed lockdowns, and expanded vaccine access. The third survey (November 1–December 20, 2021) occurred amidst mask mandates, vaccine passports, and vaccine booster doses.

### 2.3 Population and recruitment strategy

Eligible participants were a convenience sample (20) of self-identified Métis individuals aged ≥16 residing in Alberta, recruited via email to registered MNA citizens and targeted posts on the MNA’s Facebook and Twitter pages. The posts outlined the survey’s purpose, eligibility, and included a secure link. Though broadly accessible, outreach focused on individuals closely connected to the MNA, limiting non-Métis participation. After reviewing an information letter, participants provided consent and completed a web-based REDCap questionnaire, piloted before launch. Each wave included a $100 gift card draw. Responses were collected via REDCap (University of Alberta); no personal identifiers were used. Data were encrypted, securely stored, and access was MNA-governed.

### 2.4 Survey design and content

The *Misi Yehewin* surveys were administered on a single scrolling page to reduce loading time and fatigue. Each wave included items on sociodemographic characteristics, COVID-19 experiences, physical and mental health, health behaviours, community support, racism, cultural identity, and economic conditions. While core content remained consistent, item counts increased across waves: 69 (Wave 1), 78 (Wave 2), and 95 (Wave 3). Branching logic determined item visibility and reduced burden; a subset of 66 items was repeated across waves, with 28 included in the current analysis (Supplementary Table S1). Because the analysis focused on comparisons across waves, we restricted variables to items that were collected in all three waves. Some items (e.g., COVID-19 vaccination) were included only in one of the waves and were not analyzed.

Survey items were adapted from validated general and Indigenous-specific tools and reviewed by MNA team members for cultural relevance, clarity, and alignment with Métis community priorities, with wording and response options refined through discussion and agreement before each survey wave was launched. Likert-type formats and adaptive branching logic were used. Completion time was ~25 min. Responses were not editable and completeness checks were not enforced. All items included non-response options (e.g., “Prefer not to say”). To ensure privacy, no IP tracking or cookies were used. Full questionnaires are available upon request.

### 2.5 Study outcomes

Analyses focused on three domains: self-reported COVID-19 events, health and wellbeing, and social, cultural, and economic wellbeing. COVID-19 events included self-reported symptoms, testing, positive results, and hospitalizations. Health and wellbeing outcomes included perceived changes in health and quality of life and symptom screening for depression (Patient Health Questionnaire-2 [PHQ-2] ≥ 3) (21), anxiety (Generalized Anxiety Disorder-7 [GAD-7] ≥ 3) (22), and stress (Perceived Stress Scale-4 [PSS-4] ≥ 6) (23). Health behaviors encompassed reductions in physical activity, outdoor leisure time, and harvesting, hunting, or gathering, along with poorer sleep quality, and increases in screen time, and fast-food consumption. Social, cultural, and economic wellbeing indicators included time with family, land-based and cultural activities, virtual interactions, community support, cultural identity, experiences of discrimination and racism, food insecurity, and financial strain.

### 2.6 Statistical analysis

View and participation rates were not tracked as email and social media links prevented visitor identification. Completion rate was calculated as page submissions among those who initiated the survey. Sociodemographic characteristics were summarized using frequencies and percentages. Variables included age group (16–24, 25–64, 65+), gender, relationship status, income, education, employment, and residence. Differences across waves were tested using Chi-square tests, excluding missing values. For adjusted analyses, we initially considered all measured sociodemographic characteristics as potential covariates. Those that differed significantly across waves (*p* < 0.05) –age group, gender identity, household income tertile, and employment status– were retained in all regression models to account for differences in sample composition across waves.

Outcomes were summarized by wave using frequencies and proportions (expressed as percentages). Response categories were collapsed (e.g., “increased a lot” and “increased a little”) and dichotomized to reflect positive or negative experiences. The Jonckheere–Terpstra test (24) was used to assess whether there were ordered sample-level differences in each outcome across the three survey waves (statistical significance set at *p* < 0.05). This non-parametric test is appropriate for detecting ordered differences across independent groups (24). Based on the direction of wave-specific estimates, outcomes were classified as higher in later waves, lower in later waves, or similar across waves. Outcomes with statistically significant differences across waves were further analyzed using logistic regression models adjusted for age, gender identity, household income, and employment to account for differences in covariate distributions across waves. Predicted proportions from these models were used to estimate adjusted percentage differences between waves, with Bonferroni-adjusted 95% confidence intervals (CI) reported. As samples were independent across waves, results do not reflect individual-level changes. Missing data were not imputed; analyses used complete case observations. We estimated age-related sampling bias by comparing the sample age distribution with the 2021 census data (Supplementary Tables S2, S3), and conducted age-stratified analyses for age groups 16–24, 25–64, and ≥65 years as a sensitivity analysis (Supplementary Tables S4–S7), with exact cell counts <10 suppressed to preserve confidentiality. All analyses were conducted using Stata (Release 18; StataCorp LLC, College Station, TX).

## 3 Results

### 3.1 Sample and sociodemographic characteristics

**Figure 1:**
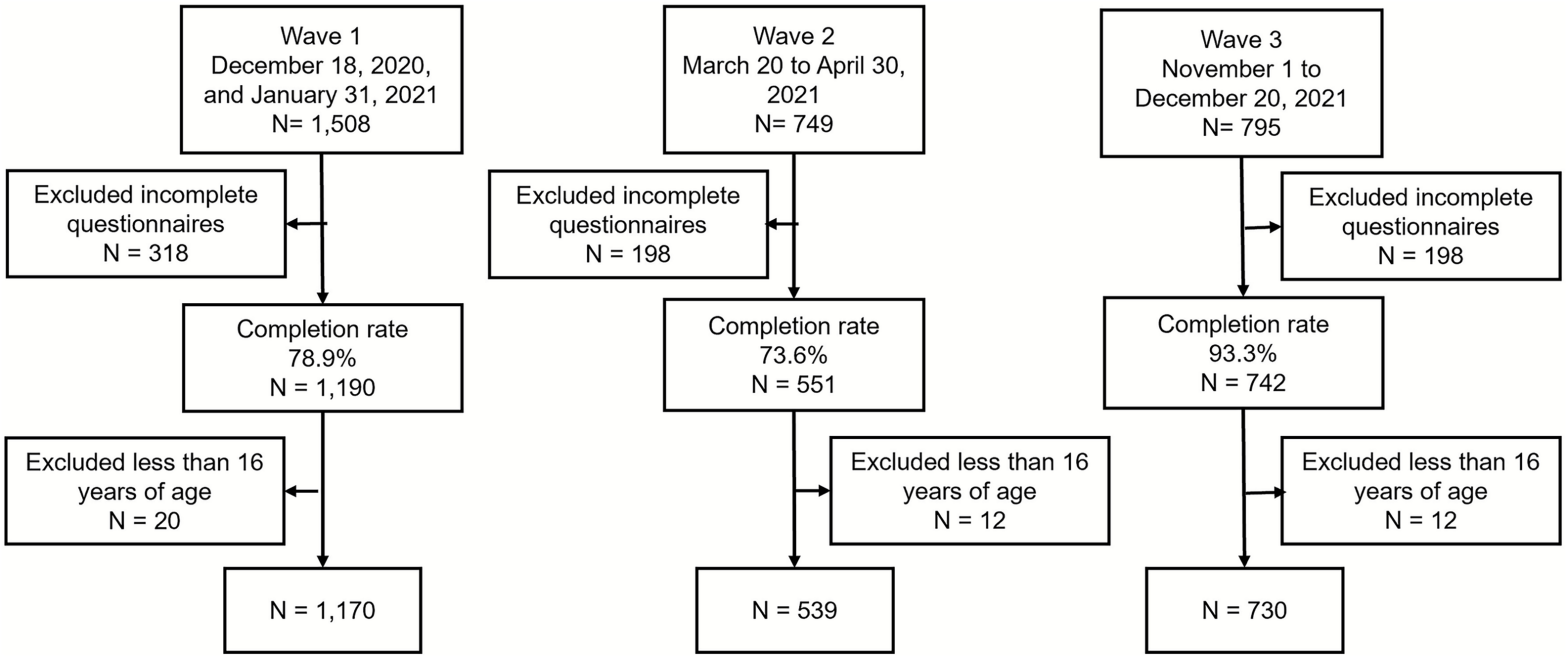
Flowchart of survey responses.

A total of 3,052 responses were received across the three survey waves: 1,508 in Wave 1, 749 in Wave 2, and 795 in Wave 3. After excluding 613 incomplete or ineligible responses, the final analytical sample included 2,439 participants: 1,170 in Wave 1, 539 in Wave 2, and 730 in Wave 3 (Figure 1). Sociodemographic characteristics are shown in Table 1. Most respondents were aged 25–64 and identified as women. Compared to earlier waves, Wave 3 included fewer older adults (*p* < 0.05), more women (p < 0.05) and a higher proportion of participants in the lowest income tertile (p < 0.05). Employment status also differed significantly across waves, with fewer students and more full-time homemakers in Wave 3.

**Table 1:** Sociodemographic characteristics across survey waves.

Variable	Wave 1 N = 1,170	Wave 2 N = 539	Wave 3 N = 730	Total N = 2,439
	% (*n*)	% (*n*)	% (*n*)	% (*n*)
Age ^a^				
Youth (16–24)	5.0 (58)	6.1 (33)	5.5 (40)	5.4 (131)
Adults (25–64)	82.1 (961)	82.8 (446)	82.7 (604)	82.5 (2,011)
Seniors (65 +)	10.9 (128)	7.61 (41)	5.8 (42)	8.7 (211)
Missing	2.0 (23)	3.5 (19)	6.0 (44)	3.5 (86)
Gender identity ^a^				
Woman	66.1 (773)	70.9 (382)	70.8 (517)	68.6 (1,672)
Man	29.4 (344)	23.6 (127)	21.0 (153)	25.6 (624)
Two-Spirit/Other	1.5 (17)	1.1 (6)	1.4 (10)	1.4 (33)
Missing	3.1 (36)	4.5 (24)	6.9 (50)	4.5 (110)
Relationship status				
In a relationship/Married	67.8 (793)	64.4 (347)	61.0 (445)	65.0 (1,585)
Single	16.7 (195)	18.4 (99)	18.0 (131)	17.4 (425)
Separated/Divorced/Widowed	10.8 (126)	11.9 (64)	11.9 (87)	11.4 (277)
Missing	4.8 (56)	5.4 (29)	9.2 (67)	6.2 (152)
Income tertile ^a^				
Bottom third	27.8 (325)	23.9 (129)	23.0 (168)	25.5 (622)
Middle third	31.2 (373)	34.7 (187)	26.0 (190)	30.8 (750)
Top third	25.9 (303)	6.7 (36)	5.6 (41)	15.6 (380)
Missing	14.4 (169)	34.7 (187)	45.3 (331)	28.2 (687)
Education				
Primary/elementary	1.2 (14)	1.7 (9)	1.5 (11)	1.4 (34)
Secondary/High school	30.5 (357)	28.2 (152)	29.2 (213)	29.6 (722)
Read Seal/Trades cert.	12.4 (145)	10.6 (57)	9.6 (70)	11.2 (272)
College/University	42.7 (500)	44.0 (237)	42.6 (311)	43.0 (1,048)
Graduate or Professional	9.3 (109)	9.7 (52)	8.5 (62)	9.1 (223)
Missing	3.9 (45)	5.9 (32)	8.6 (63)	5.7 (140)
Employment ^a^				
Employed	55.9 (654)	57.1 (308)	53.4 (390)	55.4 (1,352)
Unemployed	15.6 (182)	17.6 (95)	11.4 (83)	14.8 (360)
Student	8.2 (96)	7.4 (40)	4.9 (36)	7.1 (172)
Homemaker full time	5.2 (61)	5.2 (28)	6.9 (50)	5.7 (139)
Retired	11.3 (132)	8.2 (44)	7.3 (53)	9.4 (229)
Missing	3.9 (45)	4.5 (24)	16.2 (118)	7.7 (187)
Area of residence				
In a city	55.2 (646)	54.0 (291)	51.0 (372)	53.4 (1,309)
In a small town	20.0 (234)	20.6 (111)	22.6 (165)	20.9 (510)
In a rural area	16.8 (197)	16.7 (90)	12.7 (93)	15.6 (380)
In a remote area	0.5 (6)	1.5 (8)	1.0 (7)	0.9 (21)
Missing	7.4 (87)	7.2 (39)	12.7 (93)	9.0 (219)

^a^Statistically significant difference (*p* < 0.05) across survey waves based on Chi-square test.

### 3.2 COVID-19 events

Experiences with COVID-19 differed across survey waves corresponding to different phases of the pandemic (Table 2). The proportion of participants who reported experiencing symptoms was 3.8% in Wave 1 and 20.6% in Wave 3, (adjusted difference: 14%; 95% CI: 8, 19%). Attempts to get tested were reported by 54.5% of participants in Wave 1 and 64.2% in Wave 3 (adjusted difference: 8%; 95% CI 2, 14%). Confirmed positive test results were reported by 5% of participants in Wave 1 and 15.1% in Wave 3 (adjusted difference: 6%; 95% CI: 1, 11%). Hospitalizations were rare: fewer than 10 participants in Waves 1 and 2 reported being hospitalized due to COVID-19, with 2.3% in Wave 3. Age-stratified analyses of COVID-19 experiences (Supplementary Table S4) were broadly consistent with the main, particularly for participants aged 25–64 years. In strata with small cell counts (<10), some outcomes (e.g., COVID-19 symptoms and positive test results) and imprecise estimates and between-wave differences were not assessed.

**Table 2:** Self-reported COVID-19-related events, and health and wellbeing outcomes during the COVID-19 pandemic.

Outcomes	Survey series			Weighted %	Results^a^	Adjusted differences		
	W1% (n/valid N)	W2% (n/valid N)	W3% (n/valid N)			W1 to W2^b^ % (95%CI)	W2 to W3^b^ % (95%CI)	W1 to W3^b^ % (95%CI)
COVID-19-related events								
COVID-19 symptoms	3.8 (41/1,074)	13.5 (64/474)	20.6 (124/602)	10.7	↑	+9 (4, 14)	+5 (−2, 11)	+14 (8, 19)
Attempts to get tested	54.5 (620/1,137)	55.4 (286/516)	64.2 (417/649)	57.5	↑	0 (−7, 7)	+8 (−3, 16)	+ 8 (2, 14)
Positive test results	5.0 (30/601)	10.5 (29/276)	15.1 (61/403)	9.4	↑	+5 (−1, 10)	+1 (−5, 7)	+6 (1, 11)
Hospital admissions due to COVID-19^c^	< 10	< 10	2.3 (15/651)	1.2	n.t	n.t	n.t	n.t
Physical and mental health								
Perceived worsening in physical health	42.4 (481/1,135)	26.5 (138/520)	25.8 (166/643)	34.2	↓	−16 (−22, −11)	0 (−7, 8).	−15 (−21, −10)
Perceived worsening in mental health	62.1 (703/1,133)	37.5 (195/520)	31.1 (201/647)	47.8	↓	−28 (−34, −22)	−4 (−11, 3)	−32 (−37, −26)
Decline in quality of life	71.1 (783/1,101)	62.0 (317/511)	54.1 (341/630)	64.3	↓	−9 (−15, −3)	−8 (−15, −1)	−17 (−23, −11)
Depressive symptoms	41.5 (450/1,085)	41.4 (205/495)	41.8 (259/619)	41.6	↔	n.t	n.t	n.t
Anxiety symptoms	44.9 (481/1,072)	46.1 (227/492)	50.3 (314/624)	46.7	↔	n.t	n.t	n.t
High perceived stress	65.3 (712/1,090)	71.4 (359/503)	71.3 (449/630)	68.4	↔	n.t	n.t	n.t
Health behaviours								
Decreased moderate-to-vigorous physical activity	41.7 (410/982)	38.9 (165/424)	31.5 (167/530)	38.3	↓	−4 (−10, 3).	−5 (−13, 2)	−9 (−16, −3)
Decreased outdoor leisure activities	41.2 (445/1,079)	38.6 (188/487)	32.9 (199/604)	38.3	↓	−1 (−8, 6)	−7 (−15, 2)	−8 (−15, −2)
Decreased harvesting, hunting, or gathering	34.9 (195/558)	35.4 (95/268)	25.6 (74/289)	32.7	↓	−2 (−12, 8)	−6 (−18, 5)	−8 (−16, −1)
Reduced sleep quality	45.7 (499/1,091)	45.7 (224/490)	42.4 (254/599)	44.8	↔	n.t	n.t	n.t
Increased screen time	76.2 (862/1,131)	58.5 (299/511)	54.0 (339/628)	66.1	↓	−21 (−27, −14)	−3 (−9, 8)	−21 (−28, −15)
Regular fast-food consumption	10.5 (118/1,125)	14.8 (74/501)	14.5 (92/633)	12.6	↔	n.t	n.t	n.t

CI, confidence interval; W, wave; n.t., not tested. ^a^ Results classification: Constant (↔) indicates no statistically significant differences in percentage across waves; (↑) and (↓) indicate statistically significant higher or lower estimates across waves, respectively. ^b^Adjusted percentage differences among survey adjusting for age, gender, income level, and employment status. Bonferroni-adjusted 95% confidence intervals (CI) are highlighted in bold when statistically significant (*p* < 0.05).

### 3.3 Physical and mental health

Perceived declines in health were most commonly reported in Wave 1 (Table 2). The proportion of participants who reported worsened physical health was 42.4% in Wave 1and 25.8% in Wave 3 (adjusted difference: −15%; 95% CI: −21, −10%). Perceptions of worsening mental health were reported by 62.1% of participants in Wave 1 and 31.1% in Wave 3 (adjusted difference: −32%; 95% CI: −37, −26%). Reports of lower quality of life were 71.1% in Wave 1 and 54.1% in Wave 3 (adjusted difference: –17, 95% CI: −23, −11%). Despite these between-wave differences in perceived wellbeing, the proportion of participants screening positive for depressive symptoms (~41%), anxiety symptoms (~47%), and high perceived stress (~68%) was similar across waves. Supplementary Table S5 presents the age-stratified results for physical and mental health, showing consistent findings across age groups, except for perceived worsening in physical health in the ≥65 age group, in which no statistically significant differences across waves were observed.

### 3.4 Health behaviours

Negative health behaviour changes were more commonly reported in earlier survey waves (Table 2). The percentage of participants reporting decreased moderate-to-vigorous physical activity was 41.7% in Wave 1 and 31.5% in Wave 3 (adjusted difference: −9%; 95% CI: −16, −3%). Similarly, spending less time on outdoor leisure activities was reported by 41.2% of participants in Wave 1 and 32.9% in Wave 3 (adjusted difference: –8, 95% CI: −15, −2%). Participation in harvesting, hunting, or gathering activities was 34.9% in Wave 1 and 25.6% in Wave 3 (adjusted difference: –8, 95% CI: −16, −1%). Increased screen time was reported by 76.2% of participants in Wave 1 and 54.0% in Wave 3 (adjusted difference: –21, 95% CI: −28, −15%). In contrast, the reporting of poor sleep quality (~45%) and fast-food consumption (~13%) was similar across waves. Stratified analyses (Supplementary Table S6) showed generally consistent patterns across age groups, except for those aged ≥65 years, where small cell counts led to imprecise estimates and limited between-wave comparisons for decreased moderate-to-vigorous physical activity and decreased outdoor leisure activities.

### 3.5 Social, cultural, and economic wellbeing

Social and cultural disruptions were more frequently reported in earlier survey waves (Table 3). The proportion of respondent who said they spent less time with family was 49.7% in Wave 1 and 30.4% in Wave 3 (adjusted difference: −19%; 95% CI: −26, −12%). Reduced participation in cultural activities was reported by 31.7% of participants in Wave 1 and 20.4% in Wave 3 (adjusted difference: −13%; 95% CI: −21, −5%). Increased virtual activity was reported by 52.4% of participants in Wave 1 and 33.0% in Wave 3 (adjusted difference: −18%; 95% CI: −31, −6%). Perceptions of community support and cultural identity remained relatively similar across waves. Food insecurity was reported by 39.4% of participants in Wave 1 and 52.9% in Wave 3 (adjusted difference: 12%; 95% CI: 6, 18%). The reporting of financial strain (~56%), witnessing racism (~54%), experiences of racism (~25%), and strong Métis identity (~89%) showed no significant differences across waves. Stratified analyses (Supplementary Table S7) showed these results were broadly consistent across age groups.

**Table 3:** Social, cultural, and economic wellbeing during the COVID-19 pandemic.

Outcomes	Survey series			Weighted %	Results^a^	Adjusted differences		
	W1% (n/valid N)	W2% (n/valid N)	W3% (n/valid N)			W1 to W2^b^ % (95%CI)	W2 to W3^b^ % (95%CI)	W1 to W3^b^ % (95%CI)
Reduced time spent with family	49.7 (509/1,024)	49.2 (243/494)	30.4 (194/639)	43.9	↓	−2 (−11, 5)	−16 (−24, −7)	−19 (−26, −12)
Reduced land-based activities	42.8 (238/556)	47.5 (135/284)	33.6 (108/321)	41.4	↔	n.t	n.t	n.t
Reduced cultural activities	31.7 (206/650)	38.2 (128/335)	20.4 (78/382)	30.1	↓	+3 (−6, 12)	−16 (−26, −6)	−13 (−21, −5)
Increased virtual activities	52.4 (205/391)	30.3 (64/211)	33.0 (66/200)	41.8	↓	−21 (−32, −10)	+ 2 (−11, 16)	−18 (−31, −6)
My community is a safe place to live in	63.3 (692/1094)	58.6 (299/510)	63.1 (393/623)	62.2	↔	n.t	n.t	n.t
There are people I can go to in my community if I have a problem	63.9 (604/946)	53.8 (253/470)	55.0 (307/558)	59.0	↓	−6 (−13, −1)	−1 (−8, 7)	−7 (−13, −1)
The local Métis community has coped well with the challenges posed by the pandemic	65.6 (516/787)	61.2 (268/438)	63.5 (325/512)	63.9	↔	n.t	n.t	n.t
I felt good about being Métis	86.8 (971/1,119)	89.3 (457/512)	91.4 (555/607)	88.6	↔	n.t	n.t	n.t
I often witnessed racism	53.8 (576/1,072)	57.8 (293/507)	52.6 (306/582)	54.4	↔	n.t	n.t	n.t
I often experienced racism	23.6 (255/1,079)	29.2 (147/504)	25.8 (151/586)	25.5	↔	n.t	n.t	n.t
Experienced food insecurity	39.4 (440/1,116)	42.6 (211/495)	52.9 (326/616)	43.9	↑	+2 (−4, 9)	+10 (3, 17)	+12 (6, 18)
Worsened financial situation	53.5 (609/1,139)	59.4 (309/520)	59.3 (381/643)	56.4	↔	n.t	n.t	n.t

CI, confidence interval; W, wave; n.t., not tested. ^a^Results classification: Constant (↔) indicates no statistically significant differences in percentage across waves; (↑) and (↓) indicate statistically significant higher or lower estimates across waves, respectively. ^b^Adjusted percentage differences among survey waves adjusting for age, gender, income level, and employment status. Bonferroni-adjusted 95% confidence intervals (CI) are highlighted in bold when statistically significant (*p* < 0.05).

## 4 Discussion

This study provides a comprehensive assessment of self-reported health and wellbeing among Métis people during the early stages of the COVID-19 pandemic. Drawing on three waves of independent samples from a cross-sectional survey series, we observed notable differences in COVID-19 cases, physical and mental health, health behaviours, and social, cultural, and economic wellbeing across key phases of the pandemic.

The higher proportion of COVID-19 cases in later survey waves aligns with worldwide and national evidence showing that Indigenous populations, including Métis, experienced disproportionately high morbidity and mortality during the pandemic (1, 25–27). In Canada, disparities in COVID-19 mortality rates were particularly evident among First Nations and Métis women, whose mortality rate was more than double that of their non-Indigenous counterparts (25).

Findings suggest that long-standing structural inequities may have influenced how Métis individuals experienced the pandemic. Although fewer participants in later survey reported perceived worsening of physical and mental health, the proportion screening positive for depressive symptoms, anxiety, and stress remained high across all three waves, indicating persistent mental health challenges. According to a systematic review published in 2023 on the impact of COVID-19 on the health and livelihoods of Indigenous peoples (26), mental health challenges were exacerbated, among other factors, by lockdown-related disruptions to essential aspects of daily life, such as food security, domestic violence, the global economic depression, and the ongoing burden of intergenerational trauma. Recent Métis-specific studies in other jurisdictions have similarly documented substantial mental health and social impacts of the pandemic, including qualitative research with Métis women, Two-Spirit, and gender-diverse people, and administrative data-linkage study with Red River Métis (12–15). These studies, highlight isolation, disruptions of cultural connection, and ongoing stressors, which align with our finding of persistent mental health challenges and reduced cultural activities across waves.

The higher reporting of experiences with food insecurity in Wave 3 compared with Wave 1 points to growing economic strain during this period, which may reflect broader vulnerabilities linked to social and economic determinants of health (1, 27, 28). These patterns highlight the importance of long-term structural approaches rather than short-term responses to address health inequities in Métis communities. The consistently high rates of reported experiences or observations of racism align with broader evidence of racist attitudes toward ethnic and racial groups in Canada during the COVID-19 pandemic (29). High reports of food insecurity, persistent racism, and perceived stress suggest that public health and social supports may not have been adequately tailored to the needs of Métis communities.

Worldwide, the scarcity of ethnically disaggregated data, particularly the absence of information on Indigenous populations, precludes a comprehensive understanding of COVID-19’s impact on these communities (26, 27). The absence of Métis-specific COVID-19 data in national surveillance systems further constrained the ability to design and implement timely responsive policies or allocate resources effectively (27). This lack of visibility in public health reporting may hinder the development of culturally appropriate and equitable interventions. In addition, consistently high levels of perceived stress, coupled with lower cultural engagement and family connections reported in later waves, highlight the potential consequences of disrupted community and cultural supports. These findings support a call for Indigenous-led mental health services grounded in cultural identity, connection to the land, and community resilience (26, 27, 30).

### 4.1 Strengths and limitations

This study has several strengths. Its cross-sectional survey series design enabled assessment of sample-level ordered differences across distinct phases of the COVID-19 pandemic. Partnership with the MNA through a formal research agreement ensured the study was culturally grounded and aligned with Métis self-determination. This collaborative approach reflects growing evidence that Indigenous-led research is key to addressing health inequities and producing actionable insights (1, 27). The pandemic highlighted the effectiveness of Indigenous-led public health responses, reinforcing calls for greater Métis governance in health policy (30). Importantly, this study fills a critical gap by generating Métis-specific data during a period when national surveillance systems largely excluded this population.

Some limitations must be considered. The convenience sample limits the generalizability, as participants may differ from the broader Métis population. We used complete case analysis and did not impute missing values because the missing-at-random assumption was unlikely to hold for variables with substantial missingness, such as income. Taken together, these factors mean that observed differences between survey waves may reflect a combination of temporal changes in the underlying population, sampling variation, cohort effects, or selection bias inherent in the convenience sampling approach. Younger Métis people, particularly those aged 16–24, were underrepresented in our sample, and we could not calculate participation rates because the number of eligible individuals at each online survey wave was unavailable. Recruitment through MNA networks and web-based platforms required internet access and basic digital skills, so individuals with limited digital literacy or connectivity may be underrepresented. Women comprised most respondents in all waves, which may limit generalizability to men and gender-diverse Métis people. To partly address these imbalances, we adjusted all models for gender and conducted age-stratified analyses, which yielded results consistent with our main findings. Estimates for outcomes that vary by age (e.g., screen time, food insecurity, and mental health) may therefore not fully capture patterns in the broader Métis population, and the direction and magnitude of this bias cannot be determined with our data.

We did not collect comparable vaccination data across all waves, in part because vaccines were not yet available in Wave 1 and were only beginning to roll out during Wave 2, so we could not assess vaccination as a potential moderator of differences in outcomes between waves. More broadly, differences between earlier and later waves may also reflect unmeasured changes in the pandemic context, including vaccination rollout, evolving public health measures, and other time varying factors that were not captured in the study. Self-reported data may be subject to recall or social desirability bias, and COVID-19 status and related events were not verified with medical records or test documentation, so some misclassification is possible. Adjusted estimates are intended to account for major sociodemographic differences in sample composition across survey waves and should not be interpreted as causal effects of pandemic phase. Finally, the cross-sectional series design prevents causal inference or tracking of individual experiences over time; findings reflect sample-level differences across three independent survey waves.

## 5 Conclusion

This study provides a timely account of Métis health and wellbeing in Alberta during the early stages of the COVID-19 pandemic. While some outcomes, such as perceived physical and mental health, were more favorable in later stages in this period, high levels of perceived stress and rising food insecurity persisted. Findings highlight the impact of structural inequities and the limitations of mainstream pandemic responses in addressing Métis-specific needs. Moving forward, Métis-led public health strategies that prioritize culturally responsive mental health and social supports are urgently needed. Future research and policy must be guided by Métis governance to ensure effective, community aligned interventions.

## Data Availability

The data supporting this research are under the custodianship of the Otipemisiwak Métis Government of the Métis Nation within Alberta (MNA) and cannot be shared publicly due to ethical and privacy considerations. Data access is governed by agreements between the MNA and research partners, ensuring that Métis health information remains secure, confidential, and used in alignment with Métis self-determination principles. These agreements prohibit public sharing of the dataset. Access may be considered for researchers who meet pre-specified criteria, subject to approval by the MNA.

## References

[1] Curtice K Choo E. Indigenous populations: left behind in the COVID-19 response. Lancet. (2020) 395:1753. doi: 10.1016/S0140-6736(20)31242-3, 32505246 PMC7272170

[2] Khanijahani A Iezadi S Gholipour K Azami-Aghdash S Naghibi D. A systematic review of racial/ethnic and socioeconomic disparities in COVID-19. Int J Equity Health. (2021) 20:248. doi: 10.1186/s12939-021-01582-4, 34819081 PMC8611382

[3] Mosnaim G Carrasquel M Wolfson AR Peters J Lang D Rathkopf M. Social determinants of health and COVID-19. J Allergy Clin Immunol Pract. (2023) 11:3347–55. doi: 10.1016/j.jaip.2023.07.027, 37507069

[4] Gracey M King M. Indigenous health part 1: determinants and disease patterns. Lancet. (2009) 374:65–75. doi: 10.1016/S0140-6736(09)60914-4, 19577695

[5] Adelson N. The embodiment of inequity: health disparities in aboriginal Canada. Can J Public Health. (2005) 96:S45–61. doi: 10.1007/BF03403702, 16078555 PMC6975716

[6] Ospina MB Rowe BH Senthilselvan A King M Stickland MK Hassen S . Epidemiological and health services indicators of chronic obstructive pulmonary disease among Métis in Alberta. Edmonton: Métis Nation of Alberta and University of Alberta (2017).

[7] National Collaborating Centre for Aboriginal Health. Determinants of the prevalence and severity of influenza infection in indigenous populations in Canada. Prince George (BC): National Collaborating Centre for Aboriginal Health (2016).

[8] National Collaborating Centre for Aboriginal Health. The 2009 H1N1 influenza pandemic among first nations, Inuit, and Métis peoples in Canada: Epidemiology and gaps in knowledge. Prince George (BC): National Collaborating Centre for Aboriginal Health (2016).

[9] Public Health Agency of Canada. Flu watch: chronology: 2009 (2013). Available online at: http://publications.gc.ca/site/eng/9.507424/publication.html (accessed March 10, 2025).

[10] Boggild AK Yuan L Low DE McGeer AJ. The impact of influenza on the Canadian first nations. Can J Public Health. (2011) 102:345–8. doi: 10.1007/BF03404174, 22032099 PMC6973748

[11] Kumar MB Wesche S McGuire C. Trends in Métis-related health research (1980-2009): identification of research gaps. Can J Public Health. (2012) 103:23–8. doi: 10.1007/BF03404064, 22338324 PMC6974209

[12] Simms AJ King KD Tsui N Edwards SA Mecredy G Nation M . COVID-19 vaccine behaviour among citizens of the Métis Nation of Ontario: a qualitative study. Vaccine. (2023) 41:5640–7. doi: 10.1016/j.vaccine.2023.07.060, 37550144

[13] Driedger SM Maier R Capurro G Jardine C Tustin J Chartrand F . “There’s a little bit of mistrust”: Red River Métis experiences of the H1N1 and COVID-19 pandemics. Risk Anal. (2024) 44:1770–87. doi: 10.1111/risa.14274, 38286593

[14] Jones C Auger MD Paul W Monchalin R. “I’m still not over feeling so isolated”: Métis women, two-Spirit, and gender-diverse people’s experiences of the COVID-19 pandemic. Can J Public Health. (2024) 115:199–208. doi: 10.17269/s41997-023-00849-3, 38231468 PMC11006636

[15] Murdock M Campbell E Durant S Couchie C Meekis C Rae C . Indigenous peoples’ evaluation of health risks when facing mandatory evacuation for birth during the COVID-19 pandemic: an indigenous feminist analysis. BMC Health Serv Res. (2024) 24:1174. doi: 10.1186/s12913-024-11489-9, 39363358 PMC11447931

[16] Statistics Canada. Membership in a Métis organization or settlement: Findings from the 2021 census of population. (2022). Available online at: https://www12.statcan.gc.ca/census-recensement/2021/as-sa/98-200-X/2021006/98-200-X2021006-eng.cfm (accessed July 17, 2025)

[17] National Aboriginal Health Organization Métis Centre. Principles of ethical Métis Research (2017). Available online at: https://achh.ca/wp-content/uploads/2018/07/Guide_Ethics_NAHOMetisCentre.pdf (accessed June 19, 2025).

[18] Sharma A Minh Duc NT Luu Lam Thang T Nam NH Ng SJ Abbas KS . A consensus-based checklist for reporting of survey studies (CROSS). J Gen Inten Med. (2021) 36:3179–87. doi: 10.1007/s11606-021-06737-1 PMC8481359 33886027

[19] Eysenbach G. Improving the quality of web surveys: the checklist for reporting results of internet e-surveys (CHERRIES). J Med Internet Res. (2004) 6:e34. doi: 10.2196/jmir.6.3.e34, 15471760 PMC1550605

[20] Tyrer S Heyman B. Sampling in epidemiological research: issues, hazards and pitfalls. BJPsych Bull. (2016) 40:57–60. doi: 10.1192/pb.bp.114.050203, 27087985 PMC4817645

[21] Kroenke K Spitzer RL Williams JB. The patient health Questionnaire-2: validity of a two-item depression screener. Med Care. (2003) 41:1284–92. doi: 10.1097/01.MLR.0000093487.78664.3C, 14583691

[22] Spitzer RL Kroenke K Williams JB Löwe B. A brief measure for assessing generalized anxiety disorder: the GAD-7. Arch Intern Med. (2006) 166:1092–7. doi: 10.1001/archinte.166.10.1092, 16717171

[23] Cohen S Kamarck T Mermelstein R. A global measure of perceived stress. J Health Soc Behav. (1983) 24:385–96. doi: 10.2307/2136404, 6668417

[24] Jonckheere AR. A distribution-free k-sample test against ordered alternatives. Biometrika. (1954) 41:133–45. doi: 10.1093/biomet/41.1-2.133, 36970824

[25] Statistics Canada. The daily study: COVID-19 mortality among first nations people and Métis in Canada, 2020 and 2021. (2024). Available online at: https://www150.statcan.gc.ca/n1/daily-quotidien/240716/dq240716b-eng.htm (accessed March 10, 2025).

[26] Pickering K Galappaththi EK Ford JD Singh C Zavaleta-Cortijo C Hyams K . Indigenous peoples and the COVID-19 pandemic: a systematic scoping review. Environ Res Lett. (2023) 18:033001. doi: 10.1088/1748-9326/acb804, 36798651 PMC9923364

[27] Mallard A Pesantes MA Zavaleta-Cortijo C Ward J. An urgent call to collect data related to COVID-19 and indigenous populations globally. BMJ Glob Health. (2021) 6:e004655. doi: 10.1136/bmjgh-2020-004655, 33653731 PMC7929635

[28] Levkoe CZ McLaughlin J Strutt C. Mobilizing networks and relationships through indigenous food sovereignty: the indigenous food circle’s response to the COVID-19 pandemic in Northwestern Ontario. Front Commun. (2021) 6:672458. doi: 10.3389/fcomm.2021.672458

[29] Fahim C Cooper J Theivendrampillai S Pham B Straus SE. Exploring Canadian perceptions and experiences of stigma during the COVID-19 pandemic. Front Public Health. (2023) 11:1068268. doi: 10.3389/fpubh.2023.1068268, 36960376 PMC10027913

[30] Richardson L Crawford A. COVID-19 and the decolonization of indigenous public health. Can Med Assoc J. (2020) 192:E1098–100. doi: 10.1503/cmaj.200852, 32958575 PMC7532009

