# Supplementary material for: Misi Yehewin (big breath): a cross-sectional survey series of Métis health and wellbeing during the early COVID-19 pandemic in Alberta, Canada
Source: Front Public Health. 2026 Jan 13;13:1741161. doi: 10.3389/fpubh.2025.1741161 (PMC12835244; doi:10.3389/fpubh.2025.1741161)
Supplement: Supplementary file 1 [file Data_Sheet_1.PDF]

# *Supplementary Material*

## Supplementary Tables

**Table S1.** Survey questions and response coding across three waves

| **Survey item** | **Health outcome item and coding** |
|---|---|
| **Experiences with COVID-19** | |
| Did you experience COVID-19 symptoms? | Experienced COVID-19 symptoms<br>Yes, if recovered, or still experienced symptoms<br>No, otherwise |
| Have you tried getting tested for COVID-19? | Tried getting tested for COVID-19<br>Yes, if I got tested or tried but did not get tested<br>No, otherwise |
| Was your test positive for COVID-19? | Tested positive for COVID-19<br>Yes,<br>No |
| Were you admitted to hospital because of COVID-19 symptoms? | Admitted to hospital due to COVID-19<br>Yes,<br>No |
| **Physical and mental health during the COVID-19 pandemic** | |
| Changes in your physical health during the COVID-19 pandemic. | Felt that physical health worsened<br>Yes, if worse<br>No, if same or better |
| Changes in your mental health worsened during the COVID-19 pandemic. | Felt that mental health worsened<br>Yes, if worse<br>No, if same or better |
| Changes in your quality of life during the COVID-19 pandemic. | Worsened quality of life<br>Yes, if worsened<br>No, if same or improved |
| Diagnostic of depressive symptoms over the past two weeks. | Depressive symptoms (past two weeks)<br>Based on the Patient Health questionnaire-2 (PHQ-2). Cut-off score >= 3.<br>Yes, if PHQ-2 score >= 3<br>No, if PHQ-2 score <= 2 |
| Diagnostic of anxiety symptoms over the past two weeks. | Anxiety symptoms (past two weeks)<br>Based on the Generalized Anxiety Disorder (GAD2) questionnaire. Cut-off score >= 3<br>Yes, if GAD-2 score >= 3<br>No, if GAD-2 score <= 2 |
| Perceived stress symptoms over the past month. | Perceived stress symptoms (past month)<br>Based on the Perceived Stress Scale-4 (PSS-4). Cut-off score >=6 (high levels of stress).<br>Yes, if PSS-4 score >= 6<br>No, if PSS-4 score <= 5 |
| **Health behaviours during the COVID-19 pandemic** | |
| Changes in moderate to vigorous effort physical activity such as brisk, walking, running, or lifting weights. | Reduced moderate-to-vigorous effort physical activity<br>Yes, if decrease<br>No, if stayed the same or increased |
| Changes in doing any leisure activities (for enjoyment) outside. | Reduced outside leisure activities<br>Yes, if decreased<br>No, if stayed the same or increased |

| Survey item | Health outcome item and coding |
|---|---|
| Changes in harvesting, hunting, and/or gathering practices. | Reduced harvesting, hunting, gathering practices<br>Yes, if decreased<br>No, if stayed the same or increased |
| Changes in quality of sleep (good sleep: 7-9 hours per night, going to bed at an appropriate time, feeling rested when you wake). | Reduced sleep quality<br>Yes, if decreased<br>No, if stayed the same or increased |
| Changes in screen time outside of work duties (screen time includes TV watching, playing video games, or playing, reading, or watching videos on a phone, tablet, or computer). | Increased screen time<br>Yes, if increased<br>No, if stayed the same or decreased |
| Regular type of food consumed during the pandemic. | Regularly consumed fast food<br>Yes, if consumed mostly fast food or take-out<br>No, if consumed mostly home-made or traditional food, harvested, hunted, or gathered food, special diet for weight loss. |
| **Social, cultural, and economic well-being** | |
| Spending time with family | Reduced time with family<br>Yes, if decrease<br>No, if stayed the same or increased |
| Land-based activities like hunting and fishing | Reduced land-based activities<br>Yes, if decreased<br>No, if stayed the same or increased |
| Cultural activities like beading, jigging, cooking | Reduced cultural activities<br>Yes, if decreased<br>No, if stayed the same or increased |
| Virtual activities (museum tours, performances) | Increased virtual activities<br>Yes, if increased<br>No, if stayed the same or decreased |
| My community is a safe place to live in during the pandemic | Yes, if agree<br>No, if disagree or neither agree/disagree |
| There are people I can go to in my community if I have a problem | Yes, if agree<br>No, if disagree or neither agree/disagree |
| The local Métis community has coped well with the challenges posed by the COVID-19 pandemic | Yes, if agree<br>No, if disagree or neither agree/disagree |
| I felt good about being Métis | Yes, if agree<br>No, if disagree or neither agree/disagree |
| I often witnessed racism | Yes, if agree<br>No, if disagree or neither agree/disagree |
| I often experienced racism | Yes, if agree<br>No, if disagree or neither agree/disagree |
| Experiences of food insecurity during the pandemic. | Experienced food insecurity<br>Yes, if at least one experience, or considered using a food bank or similar service, or having exercised harvesting rights to provide food for oneself or family.<br>No, if never. |
| Current financial situation compares to prior to the COVID-19 pandemic | Worsened financial situation<br>Yes, worsened<br>No, same or better |

## Sampling bias

Age-related sampling bias was measured as the absolute difference between age groups proportions in the convenience sample and those reported in the 2021 census data for Métis in Alberta by gender identity.[1]

**Table S2**. Métis population in Alberta by age groups for women.

| | Census data[1] | | Convenience sample | | |
|---|---|---|---|---|---|
| Women age group | Freq | % | Sample Freq. | % | Absolute Difference |
| 15-24 | 10,025 | 20.0 | 97 | 5.8 | 14.2 |
| 25-34 | 10,360 | 20.6 | 308 | 18.5 | 2.1 |
| 35-44 | 8,890 | 17.7 | 452 | 27.2 | 9.5 |
| 45-54 | 8,045 | 16.0 | 430 | 25.9 | 9.9 |
| 55-64 | 7,285 | 14.5 | 269 | 16.2 | 1.7 |
| 65 and above | 5,635 | 11.2 | 106 | 6.4 | 4.8 |
| | 50,240 | 100 | 1,662 | 100 | |

**Table S3**. Métis population in Alberta by age groups for men.

| | Census data[1] | | Convenience sample | | |
|---|---|---|---|---|---|
| Men age group | Freq. | % | Sample Freq. | % | Absolute Difference |
| 15-24 | 10,855 | 23.6 | 24 | 3.4 | 20.2 |
| 25-34 | 9,250 | 20.1 | 74 | 11.9 | 8.2 |
| 35-44 | 7,900 | 17.2 | 159 | 25.6 | 8.4 |
| 45-54 | 7,210 | 15.7 | 156 | 25.1 | 9.4 |
| 55-64 | 6,160 | 13.4 | 111 | 17.9 | 4.5 |
| >65 | 4,615 | 10.0 | 98 | 15.8 | 5.8 |
| | 45,990 | 100 | 622 | 100 | |

[1] Sampling bias was assessed based on census age strata, acknowledging that our first analytical age group encompasses ages 16–20 rather than the census-defined 15–20 interval. Data Source: Statistics Canada. Indigenous identity population by gender and age: Canada, provinces and territories, census metropolitan areas and census agglomerations. Table: 98-10-0292-01. *Geography:* Canada, Province or territory, Census metropolitan area, Census agglomeration. *Universe:* Population in private households, 2021 censuses — 25% Sample data. Métis population in Alberta. Available at: https://www150.statcan.gc.ca/t1/tbl1/en/tv.action?pid=9810029201&pickMembers%5B0%5D=4.1&pickMembers%5B1%5D=2.1&pickMembers%5B2%5D=3.4 Accessed: May 28, 2025

**Table S4** Age-stratified self-reported COVID-19-related events during the COVID-19 pandemic (sensitivity analysis)

| COVID-19-related events | Survey series | | | Weighted % | Results[a] | Adjusted differences | | |
|---|---|---|---|---|---|---|---|---|
| | W1 % (n/valid N) | W2 % (n/valid N) | W3 % (n/valid N) | | | W1 to W2[b] % (95%CI) | W2 to W3[b] % (95%CI) | W1 to W3[b] % (95%CI) |
| COVID-19 symptoms (16-24) | 5.6 (scc) | 16.7 (scc) | 15.5 (scc) | 11.1 | ↔ | n.t | n.t | n.t |
| COVID-19 symptoms (25-64) | 4.1 (37/905) | 13.6 (55/404) | 22.0 (116/528) | 11.3 | ↑ | **+9 (6, 12)** | **+9 (4, 13)** | **+17 (14, 21)** |
| COVID-19 symptoms (65 +) | 0.9 (scc) | 10.3 (scc) | 7.3 (scc) | 4.1 | ↑ | **+9 (1, 20)** | -3 (-15, 9) | +6 (-2, 15) |
| Attempts to get tested (16-24) | 51.7 (30/58) | 75.0 (24/32) | 84.2 (32/38) | 67.2 | ↑ | **+ 24 (5, 44)** | +8 (-1, 27) | **+ 32 (15, 49)** |
| Attempts to get tested (25-64) | 57.2 (546/955) | 55.0 (243/442) | 65.3 (372/570) | 59.0 | ↑ | -2 (-8, 4) | **+10 (4, 16)** | **+ 8 (3, 13)** |
| Attempts to get tested (65 +) | 35.5 (44/124) | 43.9 (18/41) | 31.7 (13/41) | 36.4 | ↔ | n.t | n.t | n.t |
| Positive test results (16-24) | 10.3 (scc) | 8.7 (scc) | 3.3 (scc) | 7.3 | ↔ | n.t | n.t | n.t |
| Positive test results (25-64) | 4.9 (26/509) | 10.2 (24/235) | 16.4 (59/360) | 9.7 | ↑ | +5 (-1, 10) | **+6 (1, 12)** | **+11 (7, 16)** |
| Positive test results (65+) | 2.3 (scc) | 17.7 (scc) | 7.7 (scc) | 6.8 | ↔ | n.t | n.t | n.t |
| Hospital admissions due to COVID-19 (16-24) | ssc | ssc | scc | 1.0 | n.t | n.t | n.t | n.t |
| Hospital admissions due to COVID-19 (25-64) | ssc | ssc | 2.3 (13/572) | 1.2 | n.t | n.t | n.t | n.t |
| Hospital admissions due to COVID-19 (65 +) | ssc | ssc | scc | 1.4 | n.t | n.t | n.t | n.t |

CI=confidence interval; W=wave; scc=small cell counts (n<10). Exact numbers for ssc are suppressed in accordance with confidentiality requirements; n.t.= not tested

[a] Results classification: Constant (↔) indicates no statistically significant differences in percentage across waves; (↑) and (↓) indicate statistically significant higher or lower estimates across waves, respectively.

[b] Adjusted percentage differences among survey adjusting for gender. Bonferroni-adjusted 95% confidence intervals (CI) are highlighted in bold when statistically significant ($p<0.05$).

**Table S5** Age-stratified self-reported health and well-being outcomes during the COVID-19 pandemic (sensitivity analysis)

| Physical and mental health | Survey series | | | Weighted % | Results[a] | Adjusted differences | | |
|---|---|---|---|---|---|---|---|---|
| | W1 % (n/valid N) | W2 % (n/valid N) | W3 % (n/valid N) | | | W1 to W2[b] % (95%CI) | W2 to W3[b] % (95%CI) | W1 to W3[b] % (95%CI) |
| Perceived worsening in physical health (16-24) | 56.9 (33/58) | 30.3 (10/33) | 31.6 (12/38) | 42.6 | ↓ | **-27 (-48, -7)** | 2.5 (-19, 24). | **-25 (-45, -6)** |
| Perceived worsening in physical health (25-64) | 43.3 (412/952) | 26.7 (119/445) | 26.0 (147/565) | 34.6 | ↓ | **-17 (-22, -12)** | 0 (-6, 5) | **-18 (-22, -12)** |
| Perceived worsening in physical health (65 +) | 28.8 (36/125) | 19.5 (scc) | 17.5 (scc) | 24.8 | ↔ | n.t | n.t | n.t |
| Perceived worsening in mental health (16-24) | 74.1 (43/58) | 57.6 (19/33) | 50.0 (19/38) | 62.3 | ↓ | **-18 (-38, -2)** | -6 (-29, 18) | **-23 (-43, -4)** |
| Perceived worsening in mental health (25-64) | 63.9 (607/950) | 37.5 (167/445) | 30.5 (173/568) | 48.2 | ↓ | **-27 (-32, -22)** | **-7 (-13, -1)** | **-34 (-39, -29)** |
| Perceived worsening in mental health (65+) | 42.4 (53/125) | 19.5 (scc) | 21.9 (scc) | 33.8 | ↓ | **-24 (-38, -9)** | 3 (-14, 21) | **-21 (-36, -5)** |
| Decline in quality of life (16-24) | 80.7 (46/57) | 71.9 (23/32) | 56.8 (21/37) | 71.4 | ↓ | -7 (-26, 11) | -17 (-39, 5) | **-24 (-43, -5)** |
| Decline in quality of life (25-64) | 70.8 (653/922) | 62.3 (273/438) | 54.9 (303/552) | 64.3 | ↓ | **-9 (-14, -3)** | **-7 (-13, -1)** | **-16 (-21, -10)** |
| Decline in quality of life (65 +) | 68.9 (84/122) | 50.0 (20/40) | 41.5 (17/41) | 59.6 | ↓ | **-18 (-35, -1)** | -9 (-30, 13) | **-27 (-44, -9)** |
| Depressive symptoms (16-24) | 59.7 (34/57) | 61.3 (19/31) | 73.0 (27/37) | 64.0 | ↔ | n.t | n.t | n.t |
| Depressive symptoms (25-64) | 42.1 (384/913) | 41.4 (176/425) | 41.1 (223/543) | 41.6 | ↔ | n.t | n.t | n.t |
| Depressive symptoms (65 +) | 27.8 (32/115) | 26.3 (10/38) | 23.1 (scc) | 26.6 | ↔ | n.t | n.t | n.t |
| Anxiety symptoms (16-24) | 61.4 (35/57) | 60.6 (20/33) | 68.4 (26/38) | 63.3 | ↔ | n.t | n.t | n.t |
| Anxiety symptoms (25-64) | 45.9 (414/903) | 47.1 (198/420) | 51.6 (292/547) | 47.8 | ↔ | n.t | n.t | n.t |
| Anxiety symptoms (65 +) | 28.6 (32/112) | 23.7 (scc) | 15.4 (scc) | 24.9 | ↔ | n.t | n.t | n.t |
| High perceived stress (16-24) | 81.5 (44/54) | 87.1 (27/31) | 92.1 (35/38) | 86.2 | ↔ | n.t | n.t | n.t |
| High perceived stress (25-64) | 66.6 (614/922) | 73.0 (316/433) | 72.1 (398/552) | 69.6 | ↔ | n.t | n.t | n.t |
| High perceived stress (65 +) | 47.4 (54/114) | 42.1 (16/38) | 40.0 (16/40) | 44.8 | ↔ | n.t | n.t | n.t |

CI=confidence interval; W=wave; scc=small cell counts (n<10). Exact numbers for ssc are suppressed in accordance with confidentiality requirements; n.t.= not tested

[a] Results classification: Constant (↔) indicates no statistically significant differences in percentage across waves; (↑) and (↓) indicate statistically significant higher or lower estimates across waves, respectively.

[b] Adjusted percentage differences among survey adjusting for gender. Bonferroni-adjusted 95% confidence intervals (CI) are highlighted in bold when statistically significant (p<0.05).

**Table S6** Age-stratified self-reported health behaviours during the COVID-19 pandemic (sensitivity analysis)

| **Health behaviours** | **Survey series** | | | **Weighted %** | **Results**[a] | **Adjusted differences** | | |
|---|---|---|---|---|---|---|---|---|
| | **W1 % (n/valid N)** | **W2 % (n/valid N)** | **W3 % (n/valid N)** | | | **W1 to W2**[b] **% (95%CI)** | **W2 to W3**[b] **% (95%CI)** | **W1 to W3**[b] **% (95%CI)** |
| Decreased moderate-to-vigorous physical activity (16-24) | 53.2 (25/47) | 27.6 (scc) | 16.1 (scc) | 35.5 | ↓ | **-28 (-49, -7)** | -9 (-29, 12) | **-37 (-56, -18)** |
| Decreased moderate-to-vigorous physical activity (25-64) | 41.9 (350/836) | 38.3 (140/366) | 31.9 (149/467) | 38.3 | ↓ | -4 (-10, 2) | **-7 (-13, -2)** | **-10 (-15, -5)** |
| Decreased moderate-to-vigorous physical activity (65 +) | 35.4 (35/99) | 58.6 (17/29) | 40.6 (13/32) | 40.6 | ↔ | n.t | n.t | n.t |
| Decreased outdoor leisure activities (16-24) | 47.3 (26/55) | 25.0 (scc) | 32.4 (12/37) | 37.1 | ↓ | **-25 (-45, -7)** | 10 (-10, 32) | -15 (-35, 5) |
| Decreased outdoor leisure activities (25-64) | 41.8 (380/910) | 39.2 (163/416) | 32.8 (174/530) | 38.6 | ↓ | -3 (-8, 3) | -7 (-13, 0) | **-9 (-14, -4)** |
| Decreased outdoor leisure activities (65 +) | 34.2 (39/114) | 44.7 (17/38) | 35.1 (13/37) | 36.5 | ↔ | n.t | n.t | n.t |
| Decreased harvesting, hunting, or gathering | 41.7 (10/24) | 25.0 (scc) | 9.1 (scc) | 29.4 | ↔ | n.t | n.t | n.t |
| Decreased harvesting, hunting, or gathering | 35.9 (171/477) | 36.3 (85/234) | 26.3 (69/262) | 33.4 | ↓ | 1 (-7, 9) | **-10 (-18, -2)** | **-9 (-16, -2)** |
| Decreased harvesting, hunting, or gathering | 24.6 (14/57) | 33.3 (scc) | 25.0 (scc) | 26.4 | ↔ | n.t | n.t | n.t |
| Reduced sleep quality (16-24) | 43.9 (25/57) | 37.9 (11/29) | 52.9 (18/34) | 45.0 | ↔ | n.t | n.t | n.t |
| Reduced sleep quality (25-64) | 47.3 (431/911) | 46.9 (197/420) | 42.5 (225/529) | 45.9 | ↔ | n.t | n.t | n.t |
| Reduced sleep quality (65 +) | 35.0 (43/123) | 40.0 (16/40) | 30.6 (11/36) | 35.2 | ↔ | n.t | n.t | n.t |
| Increased screen time (16-24) | 89.7 (52/58) | 71.0 (22/31) | 70.3 (26/37) | 79.4 | ↓ | -17 (-35, 0) | -2 (-24, 19) | **-20 (-36, -4)** |
| Increased screen time (25-64) | 77.0 (732/951) | 58.7 (257/438) | 53.6 (296/552) | 66.2 | ↓ | -18 **(-24, -13)** | -6 (-12, 7) | **-24 (-29, -19)** |
| Increased screen time (65 +) | 63.9 (78/122) | 46.3 (19/41) | 43.6 (17/39) | 56.4 | ↓ | **-20 (-37, -4)** | -1 (-21, 22) | **-20 (-37, -3)** |
| Regular fast-food consumption (16-24) | 24.6 (14/57) | 12.5 (4/32) | 26.5 (9/34) | 22.0 | ↔ | n.t | n.t | n.t |
| Regular fast-food consumption (25-64) | 10.8 (102/943) | 15.4 (66/428) | 14.7 (82/559) | 13.0 | ↔ | n.t | n.t | n.t |
| Regular fast-food consumption (65 +) | 1.6 (scc) | 10.0 (scc) | 2.5 (scc) | 3.4 | ↔ | n.t | n.t | n.t |

CI=confidence interval; W=wave; scc=small cell counts (n<10). Exact numbers for ssc are suppressed in accordance with confidentiality requirements; n.t.= not tested

[a] Results classification: Constant (↔) indicates no statistically significant differences in percentage across waves; (↑) and (↓) indicate statistically significant higher or lower estimates across waves, respectively.

[b] Adjusted percentage differences among survey adjusting for gender. Bonferroni-adjusted 95% confidence intervals (CI) are highlighted in bold when statistically significant ($p<0.05$).

**Table S7** Age-stratified social, cultural, and economic well-being during the COVID-19 pandemic (sensitivity analysis)

| Outcomes | Survey series | | | Weighted % | Results[a] | Adjusted differences | | |
|---|---|---|---|---|---|---|---|---|
| | W1 % (n/valid N) | W2 % (n/valid N) | W3 % (n/valid N) | | | W1 to W2[b] % (95%CI) | W2 to W3[b] % (95%CI) | W1 to W3[b] % (95%CI) |
| Reduced time spent with family (16-24) | 31.5 (17/54) | 40.6 (13/32) | 36.1 (13/36) | 35.3 | ↔ | n.t | n.t | n.t |
| Reduced time spent with family (25-64) | 48.1 (414/861) | 47.4 (201/424) | 29.5 (166/563) | 42.3 | ↓ | 0 (-6, 6) | **-19 (-25, -13)** | **-19 (-24, -14)** |
| Reduced time spent with family (65 +) | 71.6 (78/109) | 75.7 (28/37) | 37.5 (15/40) | 65.1 | ↓ | +4 (-12, 20) | **-38 (-59, -18)** | **-34 (-52, -17)** |
| Reduced land-based activities (16-24) | 50.0 (12/24) | 53.3 (scc) | 40.0 (scc) | 48.2 | ↔ | n.t | n.t | n.t |
| Reduced land-based activities (25-64) | 42.5 (206/485) | 48.4 (121/250) | 33.2 (97/292) | 41.3 | ↓ | +6 (-2, 14) | **-15 (-23, -7)** | **-9 (-16, -2)** |
| Reduced land-based activities (65 +) | 42.6 (20/47) | 31.6 (scc) | 35.7 (scc) | 38.8 | ↔ | n.t | n.t | n.t |
| Reduced cultural activities (16-24) | 25.8 (scc) | 40.0 (scc) | 21.4 (scc) | 29.2 | ↔ | n.t | n.t | n.t |
| Reduced cultural activities (25-64) | 31.9 (179/562) | 39.0 (115/295) | 20.6 (72/350) | 30.3 | ↓ | +8 (-1, 16) | **-18 (-27, -10)** | **-11 (-18, -4)** |
| Reduced cultural activities (65 +) | 33.3 (19/57) | 25.0 (scc) | 16.7 (scc) | 28.4 | ↔ | n.t | n.t | n.t |
| Increased virtual activities (16-24) | 65.0 (13/20) | 60.0 (scc) | 12.5 (scc) | 53.5 | ↔ | n.t | n.t | n.t |
| Increased virtual activities (25-64) | 53.8 (171/318) | 27.1 (49/181) | 33.1 (58/171) | 41.3 | ↓ | **-28 (-36, -19)** | +7 (-3, 16) | **-20 (-30, -12)** |
| Increased virtual activities (65 +) | 39.6 (21/53) | 40.0 (scc) | 41.2 (scc) | 40.0 | ↔ | n.t | n.t | n.t |
| My community is a safe place to live in (16-24) | 47.2 (25/53) | 51.5 (17/33) | 57.1 (20/35) | 51.2 | ↔ | n.t | n.t | n.t |
| My community is a safe place to live in (25-64) | 62.7 (577/921) | 58.6 (255/435) | 62.7 (343/547) | 61.7 | ↔ | n.t | n.t | n.t |
| My community is a safe place to live in (65+) | 75.0 (90/120) | 63.4 (26/41) | 73.2 (30/41) | 72.3 | ↔ | n.t | n.t | n.t |
| There are people I can go to in my community if I have a problem (16-24) | 55.6 (25/45) | 53.3 (16/30) | 46.9 (15/32) | 52.3 | ↔ | n.t | n.t | n.t |
| There are people I can go to in my community if I have a problem (25-64) | 63.7 (515/809) | 52.6 (211/401) | 54.9 (270/492) | 58.5 | ↓ | **-11 (-17, -6)** | -2 (-4, 9) | **-9 (-15, -4)** |
| There are people I can go to in my community if I have a problem (65 +) | 69.6 (64/92) | 65.8 (25/38) | 64.7 (22/34) | 67.7 | ↔ | n.t | n.t | n.t |
| The local Métis community has coped well with the challenges posed by the pandemic (16-24) | 70.5 (31/44) | 74.2 (23/31) | 71.0 (22/31) | 71.7 | ↔ | n.t | n.t | n.t |
| The local Métis community has coped well with the challenges posed by the pandemic (25-64) | 64.4 (428/665) | 59.8 (222/371) | 62.3 (281/451) | 63.9 | ↔ | n.t | n.t | n.t |
| The local Métis community has coped well with the challenges posed by the pandemic (65 +) | 73.1 (57/78) | 62.9 (22/35) | 73.3 (22/30) | 70.6 | ↔ | n.t | n.t | n.t |

| Outcomes | Survey series | | | Weighted % | Results[a] | Adjusted differences | | |
|---|---|---|---|---|---|---|---|---|
| | W1<br>% (n/valid N) | W2<br>% (n/valid N) | W3<br>% (n/valid N) | | | W1 to W2[b]<br>% (95%CI) | W2 to W3[b]<br>% (95%CI) | W1 to W3[b]<br>% (95%CI) |
| I felt good about being Métis (16-24) | 87.9 (51/58) | 81.8 (27/33) | 94.3 (33/35) | 88.1 | ↔ | n.t | n.t | n.t |
| I felt good about being Métis (25-64) | 85.9 (805/937) | 90.4 (396/438) | 90.7 (486/536) | 88.3 | ↔ | n.t | n.t | n.t |
| I felt good about being Métis (65 +) | 92.7 (115/124) | 82.5 (33/40) | 100.0 (36/36) | 92.0 | ↔ | n.t | n.t | n.t |
| I often witnessed racism (16-24) | 69.6 (39/56) | 54.6 (18/33) | 45.5 (15/33) | 59.0 | ↔ | n.t | n.t | n.t |
| I often witnessed racism (24-65) | 54.0 (485/898) | 58.4 (253/433) | 54.4 (279/513) | 55.2 | ↔ | n.t | n.t | n.t |
| I often witnessed racism (65 +) | 44.1 (52/118) | 55.0 (22/40) | 33.3 (12/36) | 44.3 | ↔ | n.t | n.t | n.t |
| I often experienced racism (16-24) | 21.8 (12/55) | 25.0 (scc) | 29.4 (10/34) | 24.8 | ↔ | n.t | n.t | n.t |
| I often experienced racism (25-64) | 24.6 (224/910) | 29.7 (128/431) | 25.6 (132/516) | 26.1 | ↔ | n.t | n.t | n.t |
| I often experienced racism (65 +) | 16.7 (19/114) | 27.5 (11/40) | 25.0 (scc) | 20.5 | ↔ | n.t | n.t | n.t |
| Experienced food insecurity (16-24) | 57.9 (33/57) | 54.6 (18/33) | 52.6 (20/38) | 55.5 | ↔ | n.t | n.t | n.t |
| Experienced food insecurity (25-64) | 39.9 (374/937) | 42.9 (181/422) | 54.1 (291/538) | 44.6 | ↑ | +2 (-3, 8) | **+11 (5, 17)** | **+13 (8, 18)** |
| Experienced food insecurity (65 +) | 27.1 (33/122) | 30.8 (12/39) | 37.5 (15/40) | 29.9 | ↔ | n.t | n.t | n.t |
| Worsened financial situation (16-24) | 58.6 (34/58) | 57.6 (19/33) | 58.3 (21/36) | 58.3 | ↔ | n.t | n.t | n.t |
| Worsened financial situation (25-64) | 55.8 (534/957) | 60.3 (269/446) | 60.0 (340/567) | 58.0 | ↔ | n.t | n.t | n.t |
| Worsened financial situation (65 +) | 33.1 (41/124) | 50.0 (20/40) | 50.0 (20/40) | 39.7 | ↔ | n.t | n.t | n.t |

CI=confidence interval; W=wave; scc=small cell counts (n<10). Exact numbers for ssc are suppressed in accordance with confidentiality requirements; n.t.= not tested

[a] Results classification: Constant (↔) indicates no statistically significant differences in percentage across waves; (↑) and (↓) indicate statistically significant higher or lower estimates across waves, respectively.

[b] Adjusted percentage differences among survey adjusting for gender. Bonferroni-adjusted 95% confidence intervals (CI) are highlighted in bold when statistically significant (p<0.05).
